# The Specialist Dementia Care Program in Australia: Evidence to date on a home‐like model of care for people with very severe behaviours and psychological symptoms of dementia

**DOI:** 10.1111/ajag.70046

**Published:** 2025-05-22

**Authors:** Mustafa Atee, Srivalli Vilapakkam Nagarajan, Rebecca Lloyd, Stephen Macfarlane, Angela Raguz, Thomas Morris

**Affiliations:** ^1^ The Dementia Centre HammondCare Osborne Park Western Australia Australia; ^2^ Faculty of Health Sciences, Curtin Medical School Curtin University Bentley Western Australia Australia; ^3^ Faculty of Medicine and Health, Sydney Pharmacy School The University of Sydney Sydney New South Wales Australia; ^4^ Centre for Research in Aged Care, School of Nursing and Midwifery Edith Cowan University Joondalup Western Australia Australia; ^5^ The Dementia Centre HammondCare St Leonards New South Wales Australia; ^6^ Faculty of Medicine and Health, Northern Clinical School The University of Sydney Sydney New South Wales Australia; ^7^ Faculty of Medicine, Nursing and Health Sciences Monash University Clayton Victoria Australia; ^8^ Faculty of Medicine and Health, Sydney School of Public Health The University of Sydney Sydney New South Wales Australia; ^9^ Faculty of Health University of Canberra Canberra Australian Capital Territory Australia; ^10^ Faculty of Medicine and Health, School of Clinical Medicine The University of New South Wales Sydney New South Wales Australia

**Keywords:** behavioral symptoms, dementia, homes for the aged, neuropsychiatry, nursing homes, residential aged care facility, subacute care

## Abstract

**Objectives:**

Behaviours and psychological symptoms of dementia (BPSD) have a serious impact on care and health outcomes, such as inappropriate pharmacotherapy and impaired quality of life. These symptoms are common across care settings but are more prevalent in residential aged care homes (RACHs). BPSD such as aggression and psychosis may pose a high risk of harm to residents, co‐residents, caregivers and families, and the severe forms of these BPSD are linked to RACH premature admission. When people with very severe BPSD in Australia cannot be cared for in mainstream RACHs, the Specialist Dementia Care Program (SDCP) is offered. This article describes the SDCP model of care and examines the available evidence on SDCP outcomes.

**Methods:**

The SDCP model of care delivers person‐centred care via multidisciplinary staff in small, ‘cottage‐like’, domestic units with a familiar, dementia‐friendly care environment for an anticipated duration of 12  months. SDCP units are designed to stabilise or reduce BPSD, facilitate transition to mainstream RACHs, prevent unnecessary hospitalisations and minimise health‐care costs.

**Results:**

Preliminary evidence suggests that SDCP units may enhance resident outcomes, decrease the severity of BPSD and improve quality of life.

**Conclusion:**

This article highlights the importance of prioritising the type of care this program provides to people with very severe BPSD.

**Practice impact**

Managing very severe BPSD is challenging and requires dementia‐friendly, person‐centred, domiciliary and multidisciplinary care. Evidence suggests that the Specialist Dementia Care Program (SDCP) may improve multiple clinical and care outcomes for residents, such as decreasing the severity of BPSD, facilitating successful transitions into mainstream RACHs and reducing reliance on psychotropic medications.

**Policy impact**

The SDCP could play a crucial role in addressing systemic challenges in dementia care, particularly in reducing hospital bed block associated with a lack of suitable care options for people experiencing very severe BPSD in Australia. Financial incentives would encourage aged care providers to build a BPSD‐focused multidisciplinary workforce. Further research and comprehensive data collection are necessary to evaluate long‐term outcomes, refine care models and inform evidence‐based policy decisions.

## 1 INTRODUCTION

Dementia is a progressive neurocognitive condition characterised by cognitive, behavioural and functional decline.^1^ Neuropsychiatric or behaviours and psychological symptoms of dementia (BPSD) are a heterogeneous group of non‐cognitive symptoms such as agitation, anxiety, depression, apathy, psychosis and sleep/appetite changes.^1^, ^2^ Very severe BPSD generally refer to harmful or disruptive behaviours that may interfere with care and cause serious physical or psychological danger to both the individual and others (e.g. caregivers) and require highly skilled staff and around‐the‐clock medical support to ensure safe and high‐quality care.^1^, ^3^, ^4^ Managing these symptoms often requires an immediate sophisticated response and coordinated multidisciplinary intervention(s) across various aged and health‐care services, including home care, residential aged care homes (RACHs) and hospitals.

There is no precise estimate on the prevalence of high‐severity BPSD. However, Brodaty *et al.* estimated that approximately 1%–10% of people with dementia experience high‐severity BPSD.^1^ Poor care delivery can cause BPSD such as severe aggression that poses a high risk of harm.^5^ These high‐risk BPSD indicate the dangerous impact of very severe BPSD and the associated high risk of harm to self, co‐residents and caregivers.^1^ Mainstream RACHs may not be well equipped to manage this level of severity safely and to provide optimal quality care to people with very severe and/or intractable BPSD (i.e. behaviours that have not reduced after adequate trials of management e.g. non‐pharmacological interventions).^1^, ^4^, ^6^, ^7^

Uncontrolled BPSD result in a significant cost burden on the health‐care system. BPSD are a leading cause of RACH placement and care disruption and contribute to approximately 30% of dementia care costs (including avoidable hospitalisations and inappropriate prescribing).^8^ Research has shown that high‐care residents, including those with dementia, experience frequent and prolonged hospitalisations.^9^ In Australia, Gnanamanickam *et al.* estimated that the annual aged care and hospitalisation costs for residents with dementia to be 93% and 48% of the total whole‐of‐system costs (~AU$88,000 per person).^10^ It is therefore desirable to prevent any avoidable hospitalisations, particularly for those with high‐risk BPSD.^11^

The current health‐care system in Australia often struggles to cope with managing people with high‐risk BPSD. People with severe forms of BPSD frequently experience inadequate care, facing rejection from RACHs or bouncing around the health‐care system (e.g. frequent RACH relocations or hospital (re‐)admissions).^12^ The Australian Royal Commission into Aged Care Quality and Safety highlighted the systemic inadequacies in supporting individuals with very severe, high‐risk BPSD, including unsuitable physical and social environments that exacerbate these symptoms.^13^ There is a clear need for specialised models of residential care that focus on individualised and competent care for people with very severe, high‐risk and intractable BPSD.^1^, ^4^, ^6^ For example, accessing person‐centred and culturally appropriate care under well‐trained RACH staff is critical to achieve quality care for culturally and linguistically diverse people with BPSD.^14^, ^15^ Therefore, a care environment that supports whole‐of‐person needs and preferences is paramount.

Besides Australia, dementia‐friendly care environments exist in various countries. Examples that provide long‐term specialised care for people with dementia include the Hogeweyk village in the Netherlands and The Eden Alternative and the Green House Project in the United States.^16^ There is evidence that purpose‐built, dementia‐friendly care environments that utilise psychosocial, multidisciplinary, person‐centred models of care can improve quality of life for individuals experiencing intractable and severe BPSD.^7^, ^17^ In Australia, following the recommendations of the Royal Commission into Aged Care Quality and Safety, the National Aged Care Design Principles and Guidelines have been released to promote accessible and dementia‐friendly design standards for all RACHs.^13^ In 2016, the Australian Government announced the Specialist Dementia Care Program (SDCP) to transition, accommodate and support people with very severe BPSD who cannot be adequately cared for in mainstream aged care. To date, there is limited literature on these SDCP units (SDCPUs). Thus, the objectives of this article were to describe SDCPUs, their model of care and evidence related to SDCP outcomes.

## 2 DESCRIPTION OF THE AUSTRALIAN SDCP MODEL OF CARE

The Australian Government funds the SDCP with the intent to provide accommodation, care and improve the quality of life for people experiencing very severe BPSD.^1^ The key goals of the program are as follows: (a) providing best‐practice care to people with high severity BPSD, with minimal reliance on physical or chemical restraints and in a dementia‐friendly setting; (b) reducing and stabilising BPSD within 12  months (on average) to improve quality of life; and (c) facilitating the transition of these individuals back into a more stable care environment, such as a mainstream RACH.^7^

The SDCP model of care relies on four key underlying principles: (1) providing person‐centred care; (2) multidisciplinary support; (3) highly competent staff and a favourable staff to patient ratio; and (4) small, ‘cottage‐like’, domestic and familiar, dementia‐friendly care environments.^7^

Person‐centred care is proven to be beneficial to people with varying severity grades of BPSD.^18^ Person‐centred care principles affirm the value of the individual regardless of their degree of cognitive impairment, including tailoring care based on the individual's preferences, needs and background and providing a supportive social environment.^18^ Adopting these principles can reduce, for example, agitation and depression in residents of RACHs and can improve quality of life and cognitive function.^18^ Practical examples of person‐centred care include learning about residents' personal history, preferences, hobbies, food choices and provision of a familiar environment that has personal meaning/connections.^18^, ^19^

Multidisciplinary team support is a comprehensive approach of care provision to people with dementia, their families and caregivers.^20^ This involves the collective input of diverse health‐care professionals (including but not limited to geriatricians, general practitioners, nursing staff, psychiatrists, pharmacists, occupational therapists, psychologists and social workers) to address the complex and multifaceted (physical, emotional, social and cognitive) needs of people with dementia through holistic and person‐centred care.^20^

Dementia‐friendly environments empower, support and include people with dementia and their caregivers, as well as acknowledge their rights, maximise their potential and independence and maintain their dignity.^13^ For example, time spent outdoors may improve sleep and agitation, thereby improving quality of life and reducing depression for people with dementia.^21^ Careful planning and design of RACHs are required to promote independence and compensate for any experienced disabilities (e.g. toilet doors are distinctive in style and colour from other doors, key fittings have appropriate tonal contrast and lighting).^22^

**FIGURE 1:**
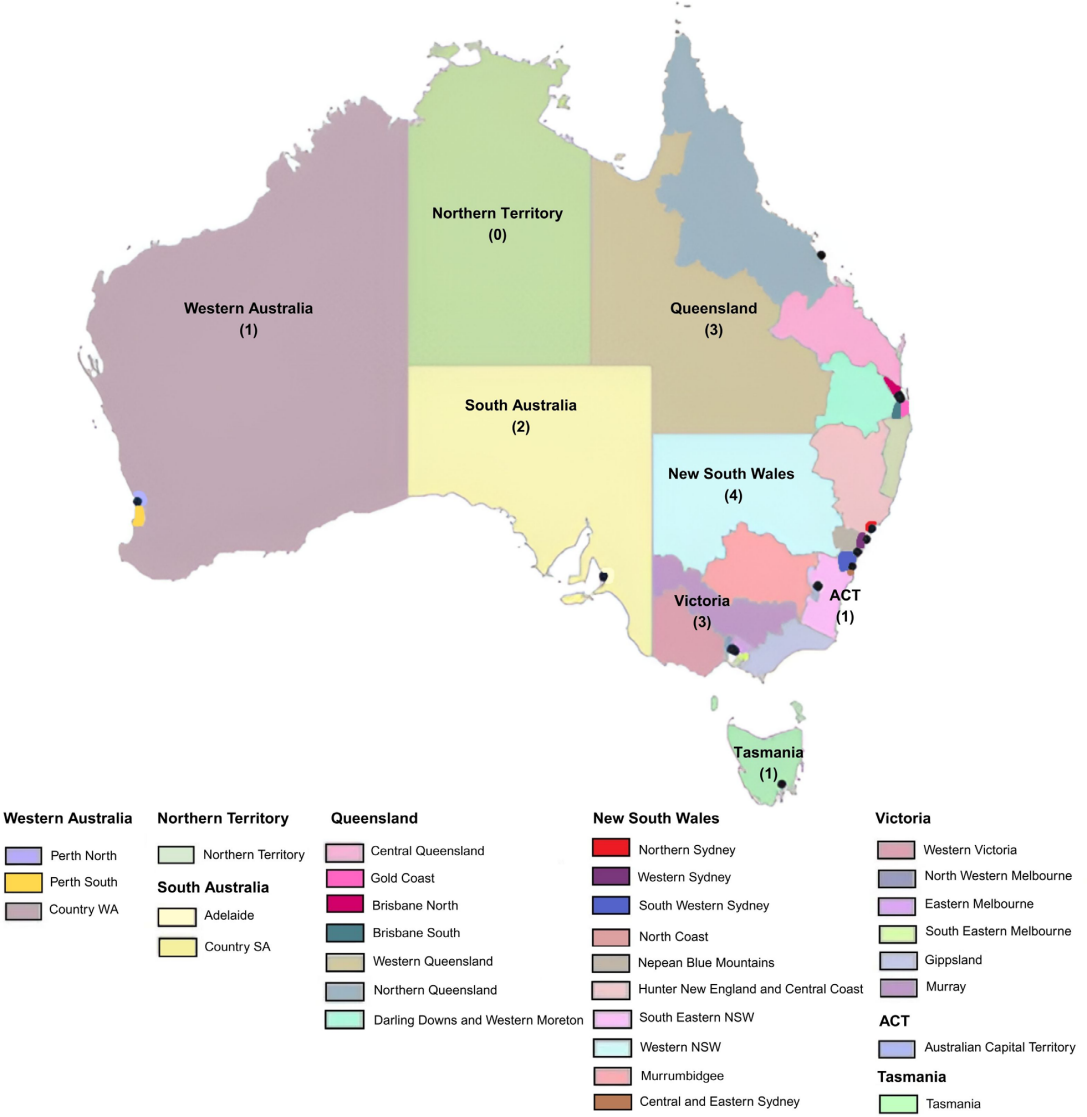
Specialist Dementia Care Program Unit (SDCPU) locations in Australia by Primary Health Networks (PHNs). The dots on this map symbolise the quantity of SDCP units (SDCPUs) within each state and territory of Australia. Due to clustering of specific dots on the map, the corresponding quantity is also indicated within parentheses. The map is accompanied by colour‐coded legends illustrating the boundaries of the Primary Health Networks (PHNs).

As of December 2024, Australia has 15 operational SDCPUs totalling 135 beds that are distributed across states and territories: New South Wales (*n* = 4), Victoria (*n* = 3), Queensland (*n* = 3), South Australia (*n* = 2), Australian Capital Territory (*n* = 1), Western Australia (*n* = 1) and Tasmania (*n* = 1).^7^ The Australian Government aims to establish a total of 35 SDCPUs across the nation with at least one unit in each of the 31 Primary Health Networks (PHNs; Figure 1).^7^ PHNs are independent bodies that receive funding from the Australian Government for the management of health‐care regions.^23^ The objectives of the PHNs are to improve the accessibility, efficiency and effectiveness of health services, focusing on individuals at high risk of adverse health outcomes.^23^

### 2.1 Unique features of the SDCP: Evidence‐based design

The distinctive features of the SDCP have been largely inspired by previous literature on successful outcomes of home‐like, small RACHs with multidisciplinary competent staff practising person‐centred care and delivering care satisfaction for residents and their families. For example, the significance of place (e.g. built environment) emphasises the therapeutic effects of sites such as home environments in the care of people with dementia.^22^ Morgan and Stewart (2002) proposed that social density (number of residents), spatial density (space per resident) and privacy (single‐occupant/private rooms) are key elements for designing specialised dementia care units that support person‐environment interactions.^24^ Evidence suggests that individuals residing in large RACHs (i.e. those with high social and spatial densities) demonstrated higher levels of aggression and anxiety compared to those in smaller RACHs.^25^ Smaller RACHs can offer a less overwhelming environment for residents with cognitive impairments or dementia.^26^ Individuals residing in small RACHs may require less support with activities of daily living, are more socially engaged and are less likely to be prescribed psychotropic medications and use physical restraints.^27^ Small RACHs can increase quality of life, reduce negative affect (e.g. depression), improve social relationships, reduce hospitalisations and increase engagement in activities for residents.^27^ Staff in small RACHs can establish better person‐centred relationships with residents, ensuring a sense of security, trust and equality.^25^ Smaller RACHs tend to have reduced staff turnover, which can lead to better continuity of care and stronger relationships between staff and residents.^28^ An Australian study found that a small, clustered domestic RACH model (compared with standard RACHs) resulted in not only fewer hospitalisations and emergency department presentations but also improved quality of life for residents without increasing operating costs.^17^ This model of care typically comprises 15 or fewer residents, outdoor areas, dedicated staff allocation to specific living units and residents' participation in meal preparation.^17^

The Residents' Experience Survey (2024) in Australian RACHs found residents with cognitive impairment and those with higher levels of behavioural support had lower satisfaction with care, particularly staff competency and safety.^29^ Compared to smaller RACHs (1–40 residents), this dissatisfaction was even more pronounced in larger RACHs (110 or more residents), which had fewer staff and limited care time.^29^

In a 2‐year longitudinal study, the small‐house nursing home model was found to improve the quality of life for residents.^27^ Buildings that offered familiarity, a range of private and communal spaces, amenities and opportunities for participation in domestic activities were linked to a higher quality of life.^27^ Smaller units may create a familiar home‐like environment that supports optimal care for people with dementia.^30^ Other key factors that facilitate quality care in RACHs include staff‐to‐resident ratio, staff competence and the availability of support services (e.g. allied health).^30^

**FIGURE 2:**
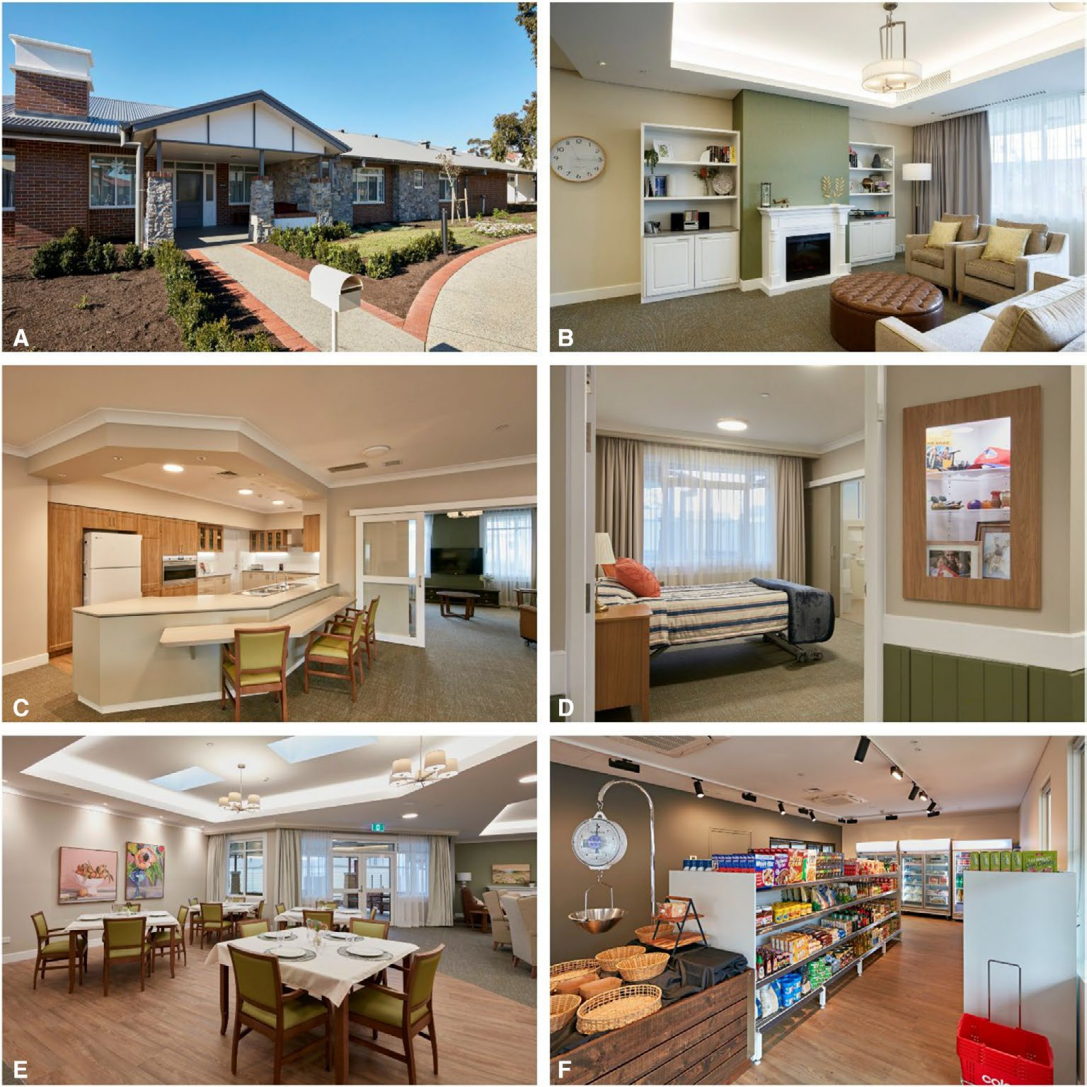
The layout of one of the cottages and one of the community facilities (corner store) at one of the SDCP providers in South Australia. The home‐like cottages accommodate typically nine beds, and include single rooms with ensuites, a domestic‐style kitchen in the centre of the home, a dining area, large communal and smaller private living areas with safe access to private and public outdoor areas and hidden utilities. (A) Cottage entrance, (B) Communal living area, (C) Kitchen, (D) Bedroom with ensuite, (E) Dining area, (F) Corner store. Images are reprinted with permission from the source.

**FIGURE 3:**
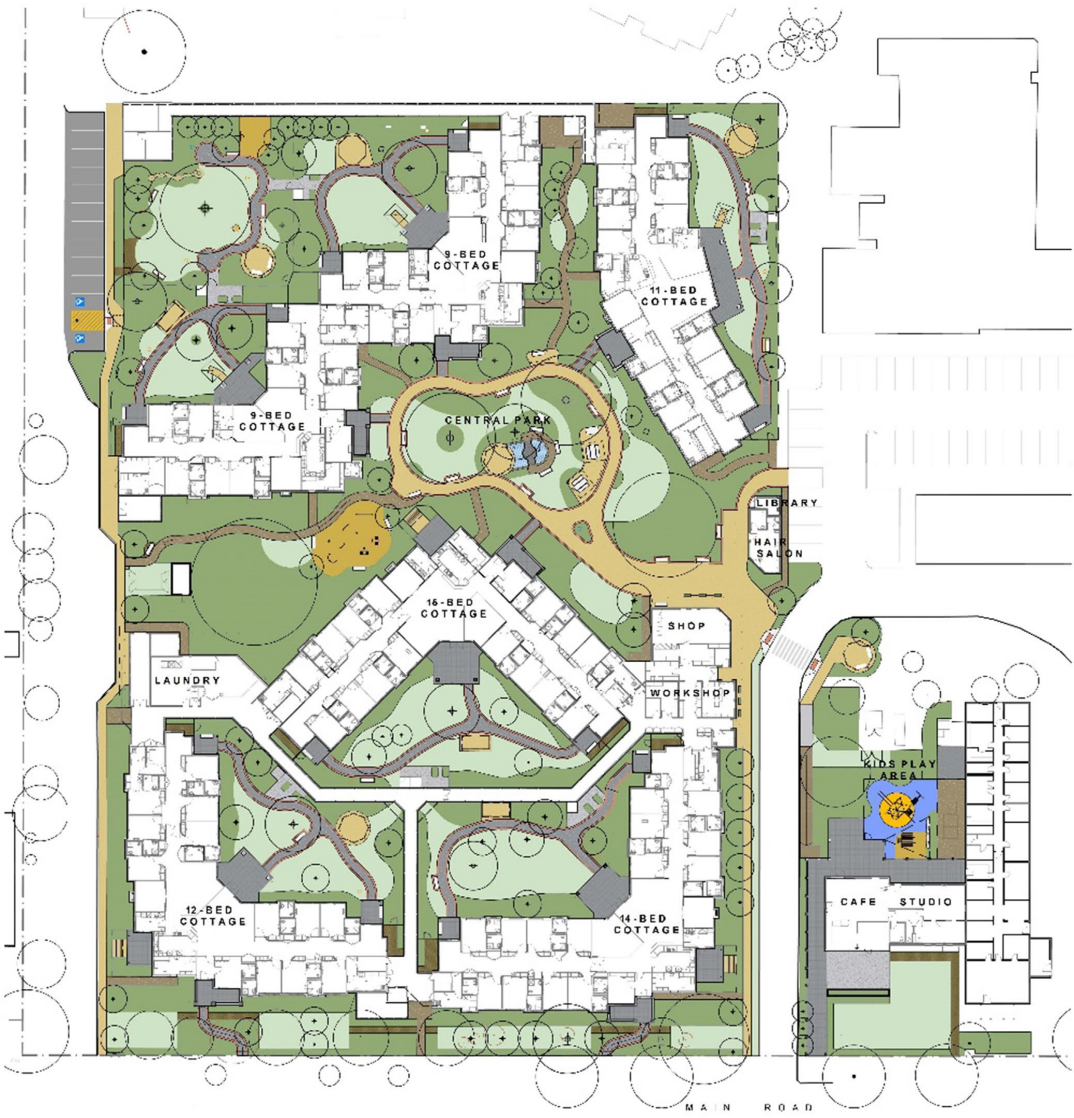
Dementia village neighbourhood facility map for one of the SDCP providers in South Australia. A neighbourhood of six cottages around a central park and amenities (e.g. hair salon, corner store, exercise park, children's playground and café). Three cottages are dementia‐specific (acute care to non‐ambulant) and two are part of the SDCP. The two nine‐bed cottages are linked to another 11‐place step‐down cottage (a post‐SDCP transition facility that aims to reduce the rate of re‐admissions). Images are reprinted with permission from the source.

Home‐like, familiar and open‐plan design characterising SDCPUs provides an effective way to enable person‐centred care. ‘Cottage‐like’ or home‐like environments include facilities offering residents the comfort and safety of a traditional, less institutionalised family residence, whilst maximising independence. For instance, the ‘cottage‐like’ respite model has been perceived by informal caregivers as a more homely, intimate, flexible and familiar service that is preferential compared to many RACHs.^31^ Families of residents often find it easier to engage with the care process in cottage‐style homes.^32^ Families may feel more comfortable and welcome in the intimate setting and have more opportunities to be actively involved in care.^32^ SDCPUs (Figure 2) are typically nine beds, offering private or semi‐private bedrooms, common living areas, wide pathways and doorways/corridors and large outdoor spaces, mirroring a residential house. ‘Cottage‐like’ homes have also shown better infection control during the COVID‐19 pandemic.^33^ See Figure 3 for a facility map of one SDCP provider in South Australia.

### 2.2 SDCP eligibility criteria, referral pathways and placement process

**FIGURE 4:**
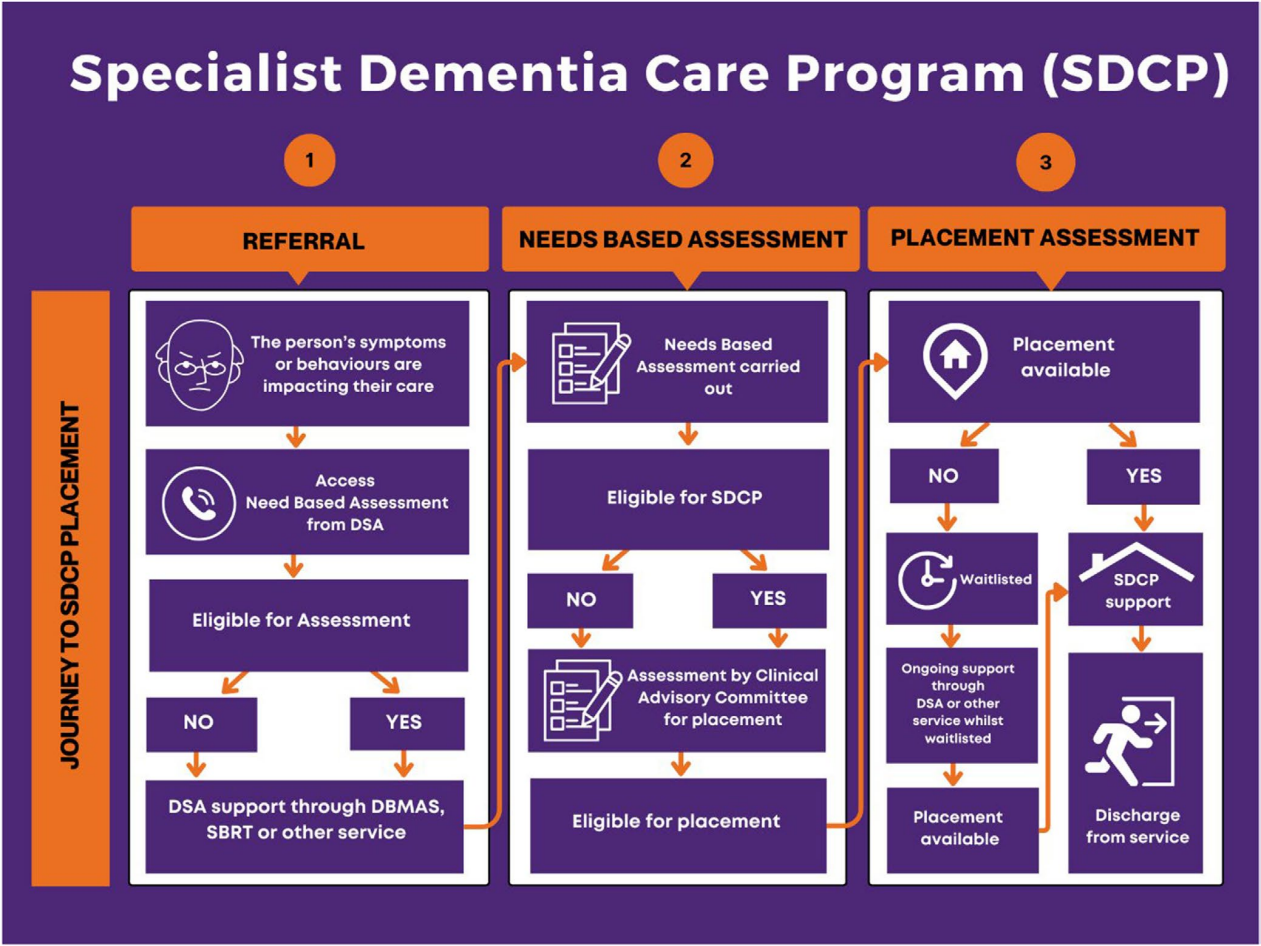
The journey from referral to placement in the Specialist Dementia Care Program (SDCP) in Australia. DSA, Dementia Support Australia; DBMAS, Dementia Behaviour Management Advisory Service; SBRT, Severe Behaviour Response Teams.

This section covers the SDCP journey, a three‐step process (Figure 4), that includes: (1) Referral; (2) Needs Based Assessment (NBA); and (3) Placement Assessment (see Table 1 for a detailed explanation of each step).

**TABLE 1:** A three‐step process from Specialist Dementia Care Program (SDCP) referral to Needs Based Assessment (NBA) and placement assessment.

Steps for SDCP placement	Detailed explanation of each step
Referral	NBA accepts referrals for individuals with very severe BPSD that impact their care for placement in the SDCPU Referrals can be made via phone, fax, or web‐based channels by individuals (e.g. families, GPs, medical specialists) or care settings (e.g. RACHs, hospitals, mental health units, and/or ACAT) Referrers are advised to review the publicly available NBA eligibility criteria and confirm whether consent for referral has been obtained from the authorised decision maker (e.g. family member) Both the referral and the referring source receive information regarding the SDCP and the assessment process If the referral is found eligible at the point of triage, an NBA assessment is conducted, if not, then they are offered alternative support through other specialised dementia programs, such as the Australian Government funded behaviour support programs Dementia Behaviour Management Advisory Services (DBMAS) or Severe Behaviour Response Team (SBRT) programs or other existing aged care services (e.g. mainstream RACH)
2 Needs based assessment (NBA)	A series of questions related to the NBA assessment process, and criteria for the SDCP are discussed with the referrer by an NBA consultant Following this discussion, referrers are requested to provide a comprehensive medico‐social history including interventions or treatment attempts to reduce BPSD, behaviour and medication charts, discharge summaries for recent hospital admissions, and a copy of consent information from the referral's authorised decision maker For eligible referrals, NBA consultants conduct an on‐site assessment (e.g. hospital or RACH) to verify that the referral meets the SDCP eligibility criteria and confirm the information obtained during the triage process Medical specialists (e.g. psychogeriatricians) discuss both eligible and ineligible outcomes and confirm the final assessment outcome If the referral does not meet the eligibility criteria for an SDCPU, the reasons for ineligibility are documented and communicated to the referrer along with the outcome, and information about alternative services (e.g. respite support). Alternatively, if the eligibility criteria are met, the referral's information is shared with a suitable SDCP provider The referral or their family can designate a preferred SDCP provider The final step in the NBA assessment involves notifying the CAC of the designated SDCP provider about the referral's eligibility
3 Placement assessment	If a place is available, SDCPU support continues until the referral is discharged from the service In instances where a place may not be immediately available, the referral is waitlisted and receives continuous support through dementia‐specific services (e.g. SBRT) until a place becomes available To assess an individual's suitability for placement in a specific SDCPU (as opposed to eligibility, determined by the NBA), the CAC of the SDCP provider considers a range of factors. These include the referral and staffing profiles, external factors (e.g. distance for families/caregivers) and the individual's specific requirements and triggers associated with their BPSD^7^ For any eligible referrals awaiting placement, DSA review occurs every 3 months until either a successful placement occurs or the eligibility criteria are no longer met A formal re‐assessment is not required within the first 12 weeks after clients have been discharged from an SDCPU

Abbreviations: ACAT, Aged Care Assessment Team; BPSD, Behaviours and psychological symptoms of dementia; CAC, Clinical Advisory Committee; DSA, Dementia Support Australia; RACH, Residential aged care home; SBRT, Severe Behaviour Response Teams; SDCPU, Specialist Dementia Care Program unit.

The SDCP eligibility requirements are as follows: (1) the individual has dementia and very severe behaviours that arise primarily from this dementia; (2) BPSD that lasted for at least 3 months; (3) other specialist services or management interventions have not been able to mitigate BPSD; and (4) the individual has undergone an Aged Care Assessment Team (ACAT) assessment, which involves a comprehensive assessment of their care needs, determines the level of care required and assists in accessing appropriate care services (e.g. RACHs).^7^ The individual is eligible for an ACAT assessment if they are aged 65 years and older (50 years or older for an Aboriginal of Torres Strait Islander person), homeless or at risk of homelessness, have complex care requirements, experience changes to family care arrangements, have had a recent fall or hospital admission and/or have cognitive impairment or dementia.

Dementia Support Australia (DSA), the national provider of dementia‐specific behaviour support programs in Australia, conducts the NBA program that evaluates the eligibility of people with BPSD for SDCPU placement.^34^ Eligibility criteria for needs based assessment include having (very) severe BPSD that occur primarily as a result of dementia and are not responding to adequate trials of other specialist services (e.g. psychogeriatric units). Consultants for NBA comprise a nationwide team of multidisciplinary professionals (e.g. specialist dementia consultants, medical specialists, registered nurses and allied health professionals (such as speech pathologists, occupational therapists, physiotherapists, social workers, psychologists, dietitians)), with significant experience working in dementia and/or aged care settings.

As part of SDCP operation, a Clinical Advisory Committee (CAC) is regularly consulted to provide advice for SDCP placement assessment, admission and post‐stabilisation discharge. Following placement, a Clinical Review Team (CRT) oversees the routine care of individuals within the SDCPU. While both the CAC and the CRT involve multidisciplinary clinicians, the CAC also has representatives from the local Primary Health Networks.

## 3 CURRENT EVIDENCE AND ASSOCIATED OUTCOMES OF SDCP‐LIKE MODELS OF CARE

To date, the existing evidence on SDCP in Australia consists primarily of two peer‐reviewed retrospective studies (Djekovic *et al.* ^35^ and Gresham *et al.* ^6^) on SDCP‐like models of care, and two independent evaluation reports (Deloitte Access Economics^4^ and the Sax Institute^36^) commissioned by the Australian Government.

Djekovic *et al.* conducted a 2‐year retrospective study (*n* = 125; 49% with high BPSD severity) at a single specialist dementia care unit within an Australian tertiary hospital.^35^ The study demonstrated that the program led to improvements in BPSD, including a reduction in BPSD severity and fewer distressing symptoms such as paranoid delusions, hallucinations and verbal agitation. This study suggested the essential role of the SDCP‐like models of care in optimising the management of severe BPSD.^35^

Gresham *et al.* conducted a 10‐year retrospective review of outcomes for 80 residents, most of whom exhibited very severe BPSD, at an aged care mental health partnership SDCP— an 8‐bed domestic‐style residential cottage with an additional ninth bedroom (step‐down approach with supported internal relocation) available for any resident transitioning to mainstream care or facing transition failure.^6^ Out of 62 admissions, only three individuals were unable to transition successfully to a mainstream RACH, with transitions occurring despite a lack of significant evidence of a reduction in behaviours. Staff perceived improvement in residents' self‐care (e.g. eating, increased social engagement). Findings demonstrated that the SDCP‐like model used in the study supports people with very severe BPSD to transition to mainstream aged care within 12  months.^6^

In 2019, the Australian Government commissioned Deloitte Access Economics, an independent economic advisory group, to conduct a mixed‐methods evaluation (phase one evaluation) of the SDCP, covering 10 SDCPUs (2019–2020) to assess the implementation, program outcomes and impact of the SDCP on people with very severe BPSD, their caregivers/families, providers and the broader health‐care system.^4^ Over a 3‐year evaluation period, 160 stakeholders were consulted via interviews, and a total of 80 survey responses from two SDCPU staff surveys involving managers, clinical nurses, care workers, allied health and in‐reach specialist clinicians were completed. Overall, there was an improvement in the quality of life and well‐being of residents, specifically in mood, communication, and interaction with family/caregivers; a reduction in BPSD (e.g. aggressive behaviour during personal care); decreased desire to abscond from the unit; and lastly, increased engagement in daily tasks and activities. Clinicians noted post‐program outcomes, such as a reduction in psychotropic medications (e.g. antipsychotics). The Deloitte evaluation recommended tracking resident progress post‐SDCP discharge to determine whether a risk‐stratified approach is necessary for transitioning high‐risk residents and to reduce future bounce‐backs or hospital admissions by implementing a step‐down discharge plan.^4^

In 2017, the Australian Government commissioned the Sax Institute to conduct a rapid review of international literature on the effective management and care of people with very severe BPSD, with the aim of informing the design and implementation of SDCPUs.^36^ This review consolidated the evidence from various international studies on the quality of care, quality indicators and outcomes, benchmarking care standards, level of services and funding support. The studies of services that effectively managed these symptoms shared eight common elements including: (1) person‐centred care; (2) a supportive physical environment; (3) specialised education, skills and training for staff and caregivers; (4) specialist medical input; (5) allied health staffing; (6) therapeutic and meaningful activities; (7) individualised assessment and care planning; and (8) a multidisciplinary approach to care.^36^ Given that this review encompassed studies of distinct international specialist dementia care models, it is advisable to exercise caution when making direct comparisons to the contemporary Australian SDCP.

The collective findings from these studies and commissioned evaluations suggest several benefits of the SDCP, including improved outcomes for residents, enhanced staff capability and confidence in caring for people with very severe BPSD, long‐term cost‐effectiveness for the Australian health and aged care system and the creation of a safe and dementia‐friendly care environment.^4^, ^6^, ^35^, ^36^

Other relevant evidence supportive of SDCP features (e.g. person‐centred care) can also be drawn from national dementia‐specific behaviour support programs in Australia, such as the Dementia Behaviour Management Advisory Service (DBMAS) for mild‐to‐moderate BPSD and the Severe Behaviour Response Teams (SBRT) for moderate‐to‐severe BPSD.^37^ Delivered by a multidisciplinary health‐care team, these programs were found effective in reducing BPSD severity and caregiver distress after applying multimodal person‐centred psychosocial interventions.^37^, ^38^

## 4 LIMITATIONS OF EXISTING RESEARCH AND FUTURE DIRECTIONS

While preliminary evidence suggests positive outcomes from the SDCP, the effectiveness of various aspects of this model of care is still poorly understood. We present several limitations and offer some recommendations for the future.

First, whilst the stand‐alone features of the SDCP (e.g. person‐centred care, multidisciplinary support) have received robust support in the literature,^18^ solid evidence to support the entire SDCP model of care is currently lacking. Longitudinal evaluations are necessary, along with consideration of more granular and meaningful outcomes for SDCPUs at the resident, staffing and organisational levels.

Second, accessibility barriers for regional, rural and remote SDCP applicants (e.g. challenges associated with transporting long‐distance residents to SDCPUs) should be considered in the proposed future planning of SDCPUs.^1^, ^4^ For example, utilising geographic demand modelling to determine priority areas for new SDCP sites. Further exploration of care needs and strategies that address sociodemographic status and equitable access for these residents is recommended.

Third, assessment of BPSD profiles should consider the differences in sociodemographics of the person and organic symptomatology of dementia subtypes. For example, aggression in frontotemporal younger‐onset dementia may be more closely associated with its neuropathological changes than with unmet needs or environmental factors.^39^ Individuals with complex medical needs or atypical dementia presentations may be unintentionally excluded. Future research should explore how BPSD‐related factors (including its fluctuating and, in some instances, intractable or resistant nature and its interaction with other comorbidities) may impact care outcomes.

Fourth, the SDCPUs are still affected by RACH operational challenges such as staffing shortages, a lack of competent staff and inadequate funding.^4^ Strategies to address workforce shortages, such as targeted recruitment, specialist training opportunities and better retention initiatives, are essential to meet residents' needs.

## 5 CONCLUSIONS

Limited yet promising preliminary evidence suggests that the SDCPUs and their features may enhance resident outcomes, decrease the severity of BPSD, facilitate successful transitions into mainstream RACHs, reduce reliance on psychotropic medications and improve quality of life.

The SDCP has the potential to play a crucial role in addressing systemic challenges in dementia care, particularly in reducing hospital bed block associated with a lack of suitable care options for people experiencing very severe BPSD. Thus, it is prudent to recognise the importance of prioritising the type of care this program provides through incentivising aged care providers to build workforce capacity that focuses on delivering quality BPSD care. Further research and comprehensive data collection are necessary to examine the evidence behind the SDCP to evaluate long‐term outcomes, refine care models, inform evidence‐based policy decisions and develop best practice guidelines for this population.

## FUNDING INFORMATION

No specific funding received for this manuscript.

## CONFLICT OF INTEREST STATEMENT

All authors are staff members of HammondCare, an independent Christian health‐care organisation that has a number of residential aged care homes in Australia and auspices the DSA programs—The Dementia Behaviour Management Advisory Service (DBMAS) and the Severe Behaviour Response Teams (SBRT). MA is a Research and Practice Lead (Team Leader) at The Dementia Centre, HammondCare. SVN is a Data and Information Officer at The Dementia Centre, HammondCare. RL is a Data and Information Analyst at The Dementia Centre, HammondCare. SM is the Head of of Clinical Services, The Dementia Centre, HammondCare. TM is the Head of Research and Information, The Dementia Centre, HammondCare. At the time of writing this article, AR is the General Manager, The Dementia Centre, HammondCare.

## Data Availability

Data sharing is not applicable as no new data were created or analysed in this article.
